# Performance of ^89^Zr-Labeled-Rituximab-PET as an Imaging Biomarker to Assess CD20 Targeting: A Pilot Study in Patients with Relapsed/Refractory Diffuse Large B Cell Lymphoma

**DOI:** 10.1371/journal.pone.0169828

**Published:** 2017-01-06

**Authors:** Yvonne W. S. Jauw, Josée M. Zijlstra, Daphne de Jong, Danielle J. Vugts, Sonja Zweegman, Otto S. Hoekstra, Guus A. M. S. van Dongen, Marc C. Huisman

**Affiliations:** 1 Department of Hematology, VU University Medical Center, Amsterdam, The Netherlands; 2 Department of Pathology, VU University Medical Center, Amsterdam, The Netherlands; 3 Department of Radiology & Nuclear Medicine, VU University Medical Center, Amsterdam, The Netherlands; National Cancer Institute, UNITED STATES

## Abstract

**Purpose:**

Treatment of patients with diffuse large B cell lymphoma (DLBCL) includes rituximab, an anti-CD20 monoclonal antibody (mAb). Insufficient tumor targeting might cause therapy failure. Tumor uptake of ^89^Zirconium (^89^Zr)-mAb is a potential imaging biomarker for tumor targeting, since it depends on target antigen expression and accessibility. The aim of this pilot study was to describe the performance of ^89^Zr-labeled-rituximab-PET to assess CD20 targeting in patients with relapsed/refractory DLBCL.

**Methods:**

Six patients with biopsy-proven DLBCL were included. CD20 expression was assessed using immunohistochemistry (IHC). 74 MBq ^89^Zr-rituximab (10 mg) was administered after the therapeutic dose of rituximab. Immuno-PET scans on day 0, 3 and 6 post injection (D0, D3 and D6 respectively) were visually assessed and quantified for tumor uptake.

**Results:**

Tumor uptake of ^89^Zr-rituximab and CD20 expression were concordant in 5 patients: for one patient, both were negative, for the other four patients visible tumor uptake was concordant with CD20-positive biopsies. Intense tumor uptake of ^89^Zr-rituximab on PET (SUV_peak_ = 12.8) corresponded with uniformly positive CD20 expression on IHC in one patient. Moderate tumor uptake of ^89^Zr-rituximab (range SUV_peak_ = 3.2–5.4) corresponded with positive CD20 expression on IHC in three patients. In one patient tumor uptake of ^89^Zr-rituximab was observed (SUV_peak_ = 3.8), while the biopsy was CD20-negative.

**Conclusions:**

This study suggests a positive correlation between tumor uptake of ^89^Zr-rituximab and CD20 expression in tumor biopsies, but further studies are needed to confirm this. This result supports the potential of ^89^Zr-rituximab-PET as an imaging biomarker for CD20 targeting. For clinical application of ^89^Zr-rituximab-PET to guide individualized treatment, further studies are required to assess whether tumor targeting is related to clinical benefit of rituximab treatment in individual patients.

## Introduction

DLBCL is an aggressive, potentially fatal, but curable form of lymphoma. It is the most common lymphoma subtype, representing 30% of all lymphoma. This malignancy develops from the B-cells in the lymphatic system and is characterized by expression of CD20, a transmembrane protein. The function of CD20 is still unknown, but as it is only expressed on B cells and not on other tissues, it is a usefull target for treatment. Rituximab, an anti-CD20 mAb, is currently incorporated in all first line and subsequent treatment regimens for DLBCL. The introduction of rituximab in first line treatment has improved the prognosis of a three-year event-free survival (EFS) from 59% to 79% for patients of 18 to 60 years old. However, patients with relapsed/refractory DLBCL have a three-year overall survival (OS) of only 49% [1]. Early relapse (<12 months) and prior rituximab treatment are associated with a worse outcome, with a three-year EFS of 21% versus 47%, suggesting rituximab resistance. Although it is standard practice to include rituximab in second line treatment, it is unclear whether individual patients benefit from repeated rituximab treatment. To obtain clinical benefit from mAb treatment tumor targeting is required. It comprises target antigen expression, as well as a drug that reaches and binds to the target.

Target expression of CD20 is assessed by IHC on a single tumor biopsy as part of the routine work up to confirm the diagnosis of DLBCL, or to confirm relapsed/refractory disease [2]. [Fluorine-18]-2-fluoro-2-deoxy-D-glucose (^18^F-FDG)-PET is incorporated in staging and response evaluation of DLBCL [3]), but provides no information on expression of CD20.

Molecular imaging with ^89^Zirconium (^89^Zr)-labeled mAbs, also known as immuno-PET, allows for visualization and quantification of tumor uptake and whole body biodistribution of ^89^Zr-mAbs [4]. Since tumor uptake depends on target expression and accessibility, it is a potential imaging biomarker for tumor targeting.

Preclinical use of ^89^Zr-rituximab has been reported [5–7] showing tracer uptake in transgenic mice with human CD20 on their B-cells. The first clinical study with ^89^Zr-rituximab reported the use of ^89^Zr-rituximab to assess radiation dose for subsequent radio-immunotherapy with ^90^Y-labeled rituximab in five patients with CD20+ B cell lymphoma [8]. However, until now no clinical study has described the correlation between CD20 expression and tumor uptake of ^89^Zr-rituximab.

For immuno-PET with other ^89^Zr-labeled mAbs two clinical trials have reported whether tumor uptake on immuno-PET and target expression in biopsies are correlated. These studies were on ^89^Zr-bevacizumab, an anti-endothelial growth factor (VEGF)-A mAb, in patients with breast cancer [9] and ^89^Zr-labeled anti-membrane-bound surface glycoprotein mesothelin (MSLN) mAb in patients with pancreatic and ovarian cancer [10]. Correlations between a measure of tumor uptake and a measure of target status were reported, to provide evidence that immuno-PET may be used as an imaging biomarker to assess tumor targeting.

The aim of this pilot study was to describe the performance of ^89^Zr-labeled-rituximab PET as an imaging biomarker to assess CD20 targeting in patients with relapsed/refractory diffuse large B cell lymphoma, by correlating tumor uptake of ^89^Zr-rituximab with CD20 expression in biopsied tumor lesions.

## Materials and Methods

Data used in this pilot study was obtained as part of an ongoing main trial (registered in the Dutch Trial Register http://www.trialregister.nl, NTR 3392) with formal ethical approval from the Medical Ethics Committee of the VU University Medical Center, Amsterdam, The Netherlands (approval date July 2012, reference 2012/165). The study was performed in compliance with the principles of the Declaration of Helsinki. All patients signed an informed consent form.

Patients with a primary diagnosis of CD20-positive DLBCL, relapsed after or refractory to first line treatment with rituximab combined with anthracycline-based chemotherapy (R-CHOP), were eligible for inclusion. A ^18^F-FDG-PET scan was performed as routine clinical staging before start of second line treatment [11].

Patients were included before start of second line treatment with rituximab combined with cisplatin-based chemotherapy. Inclusion criteria consisted of age ≥18 years, WHO performance score 0–2 and eligibility for high dose chemotherapy and autologous stem cell transplant. Patients with known central nervous system (CNS) involvement were not eligible.

Tumor biopsies were obtained to confirm refractory/relapsed disease before start of second line treatment. IHC was performed, including at least staining for CD20, CD79a, CD3 and MIB1 to support the diagnosis. As part of the routine work-up CD20 expression was reported as either present (+) or absent (-). In addition, CD20 expression was assessed semi-quantitatively as:

1. Uniformly positive in all tumor cells.
2. Heterogeneously positive, ranging from positive in the majority of cells to positivity limited to sparse groups of cells.
3. Absent, ranging from extremely sparse groups of CD20-positive cells to completely absent in all tumor cells, with a positive internal control sample and CD79a-positive.

A qualitative assessment was made for membranous or granular staining patterns. Patients were ranked based on the level of CD20 expression in order to correlate biopsy results to tumor uptake of ^89^Zr-rituximab, defined on PET images. Tumor biopsies were assessed by a pathologist blinded for immuno-PET results.

^89^Zr (T½ = 78.4 hrs) (BV Cyclotron VU, Amsterdam, the Netherlands) was coupled to rituximab via the bifunctional chelator N-succinyl-desferal. Methods used for radiolabeling have been described previously [12–14]. The radiochemical purity was assessed by instant thin layer chromatography (iTLC) and high-performance liquid chromatography (HPLC). The immunoreactive fraction was assessed by Lindmo binding assay using either Ramos (patient 1, 2, 3) or Su-DHL4 (patient 4, 5, 6) cells. The endotoxin content was determined according to pharmacopeia using an endosafe PTS reader. Sterility of each ^89^Zr-rituximab batch was assured by performing a media fill immediately after final filter sterilization of each batch and by performing a bubble-point test on the final filter used during sterile filtration. The radiochemical purity, immunoreactivity and endotoxin content of every batch were assessed prior to administration to a patient. Manufacturing and radiolabeling were performed according to Good Manufacturing Practice (GMP) standards.

Patients received a therapeutic dose of rituximab (range 700–1000 mg) on day 1 of cycle 1 of second line treatment, within 2 hours followed by ^89^Zr-rituximab (10 mg, 74 MB +/- 10%) as an intravenous bolus injection. Venous blood samples were scheduled at t = 2 hrs (D0), 72 hrs (D3) and 144 hrs (D6) post injection (p.i.). Whole blood and plasma radioactivity concentrations were measured with a gamma well counter (Wallac 1480 Wizard, Turku, Finland). Radioactivity measurements obtained with venous blood samples were correlated with image-derived blood pool measurements. Percentage injected dose (%ID) in blood pool on D6 was calculated using image derived radioactivity concentrations and total blood volume [15]. Tracer availability is commonly defined as the concentration of the tracer in the plasma fraction, therefore the total activity in plasma needs to be calculated and compared to the total activity in whole blood.

Total activity in plasma on D6 was calculated from the venous blood samples, using standard hematocrit values (0.45 for males and 0,4 for females) and total blood volume. Total activity in whole blood was calculated from the venous blood samples and total blood volume. Wilcoxon matched pairs signed rank test was used to explore a difference between both measures of total activity, since the power of the test is limited by the small sample size.

Immuno-PET scans (Gemini TF-64/Ingenuity TF-128; Philips Healthcare, Best, the Netherlands), were acquired at D0, D3 and D6 p.i., and reconstructed as described previously [16]. Each whole body immuno-PET scan was preceded by a 35 mAs low-dose computed tomography (ldCT) scan for attenuation and scatter correction. Immuno-PET scans were evaluated by a nuclear medicine physician, blinded for the ^18^F-FDG-PET and biopsy results. Lesions were considered positive in case of focal uptake exceeding local background. Immuno-PET scans were classified as positive (moderate or intense, at the D6 scan) or negative for tumor uptake of ^89^Zr-rituximab in the biopsied tumor lesion. Thereafter, the immuno-PET scans were compared with the ^18^F-FDG-PET scans to confirm tumor localization. In case of visible tumor uptake on immuno-PET, tumor volumes of interest (VOIs) were manually delineated on immuno-PET scans at D3 and D6, using a semi-automatic in-house software tool. Peak (i.e. average value of a 12mm sphere positioned within the VOI so as to obtain the highest value) and mean activity concentrations (AC_peak_ and AC_mean_, respectively) were derived per tumor VOI. For AC_mean_ standard deviations (SD) were derived per VOI. Blood pool VOIs were delineated using a fixed size VOI of the aortic arch (volume of 1.6 mL), on the ldCT and imported to the PET images. AC_mean_ was derived per blood pool VOI. Standardized uptake values (SUV_peak_ and SUV_mean_ ± SD, respectively) were calculated for tumor lesions, and decay corrected to the time of injection. Tumor to blood ratio’s (T/B) for tumor lesions were calculated as tumor AC_peak_ on D6 divided by image derived blood pool AC on D6. To assess tumor uptake over time, SUV_peak_ D6/D3 ratios were calculated.

Spearman’s rank correlation coefficient (r_s_) as well as the two-tailed p-value were calculated to explore the relation between tumor uptake of ^89^Zr-rituximab, measured as SUV_peak_ on D6, and the level of CD20 expression in the biopsied lesions. Statistical tests were performed using GraphPad Prism Version 6.02 (GraphPad Software Inc.).

## Results

Six patients with a primary diagnosis of CD20-positive DLBCL, with refractory or relapsed disease after first line treatment with R-CHOP, were included. Patient characteristics are summarized in Table 1.

**Table 1:** Patient characteristics.

**Patient**	**Gender**	**Age**	**Response to first line**	**Ann Arbor stage at relapse**	**Disease localization at relapse on** ^**18**^**F-FDG-PET**
**1**	M	23	complete remission	IV A	liver
2	F	55	partial remission	III B	cervical, para-iliac, spleen
3	M	46	complete remission	III B	supraclavicular, mediastinal, hilar, mesenteric, retro-peritoneal, para-iliac, inguinal
4	M	46	complete remission	II A	retro-peritoneal
5	M	47	complete remission	I E	nasopharynx
6	F	69	partial remission	III A	submandibular, retro-clavicular, axillar, inguinal

Patient 2 and 6 had primary refractory disease, with a partial remission (PR) after R-CHOP. The other patients had relapsed disease, of whom two patients (1 and 3) had an early relapse within 1 year after R-CHOP. In all patients ^18^F-FDG-PET scans were obtained for staging, before start of second line treatment. All patients were subsequently treated in second line with rituximab combined with high dose cytarabine, cisplatin and dexamethasone (R-DHAP).

Quality controls of ^89^Zr-rituximab were meeting the pre-set requirements. Radiochemical purity was 99.3% ± 0.2% according to iTLC and 100% according to SE-HPLC, antigen binding was ≥ 82% (Lindmo assay) and bacterial endotoxin content was <0.2 EU/mL (PTS reader) for all patients (Table 2).

**Table 2:** Quality controls of ^89^Zr-rituximab.

**Patient**	**Radiochemical purity: iTLC (%)**	**Radiochemical purity: HPLC (%)**	**Antigen binding: Lindmo assay (% binding)**	**Bacterial endotoxin content: PTS reader (EU/mL)**
Requirement	>90.0	>90.0	>70	< 2.5
1	99.2	100	92	< 0.2
2	99.2	100	82	< 0.2
3	99.4	100	83	< 0.2
4	99.2	100	87	< 0.2
5	99.6	100	91	< 0.2
6	99.2	100	93	< 0.2

Administration of ^89^Zr-rituximab was well tolerated by all patients. Four patients underwent all scans according to protocol. Due to chemotherapy induced nausea, only a scan of the head and neck area could be obtained in patient 2 at D6, and blood sampling at D3 and D6 was not feasible. Patient 6 cancelled the D3 scan due to diarrhea and nausea, most likely caused by an infectious entero-colitis. For patient 3 no venous blood sample was obtained at D3.

**Fig 1:**
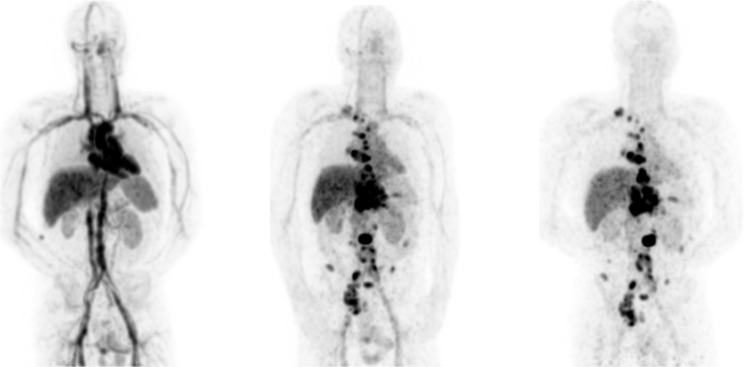
Example of whole body distribution of ^89^Zr-rituximab over time. Maximum intensity projections at D0, D3 and D6 p.i. for patient 3.

^89^Zr-rituximab in blood pool, liver, spleen and kidneys was evident at D0, decreasing over time in all patients (Fig 1). All sites of positive tumor uptake identified at D6 were also observed at D3, whereas no tumor uptake was identified on D0. At D6 on average 27.6% ± 5.7% ID of ^89^Zr-rituximab was still circulating in blood pool. For the difference between total activity at D6 as derived from plasma samples and whole blood samples a p-value of 0.38 was obtained with Wilcoxon matched pairs signed rank test (S1 Fig). Image derived and venous sampled whole blood activity concentrations were correlated with an R^2^ of 0.98 and slope of 0.85.

**Fig 2:**
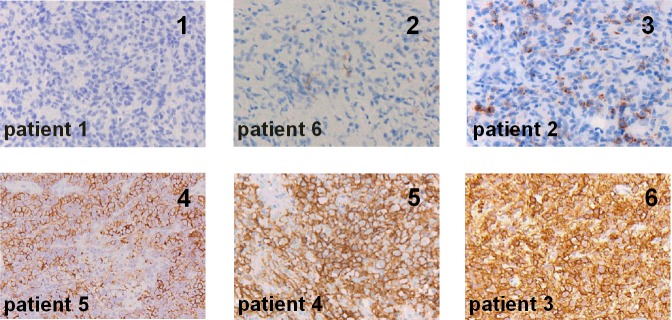
Biopsies ranked in order of increasing CD20 expression. Per panel the CD20 rank is indicated in the right upper corner and the patient number in the left lower corner. 1) Core-needle biopsy of DLBCL (liver) in patient 1 shows completely absent CD20 expression. 2) Core-needle biopsy of DLBCL (axillar lymph node) in patient 6 showing almost completely absent CD20 expression: extremely sparse groups of CD20-positive tumor cells with granular staining pattern, considered as absent CD20 expression. 3) Excision biopsy of DLBCL (cervical lymph node) in patient 2 shows heterogeneously positive CD20 expression in sparse groups of cells, granular staining pattern. 4) Excision biopsy of DLBCL (nasopharynx) in patient 5 showing heterogeneously positive CD20 expression in the majority of cells, membranous staining pattern. 5) Excision biopsy of DLBCL (retroperitoneal lymph node) in patient 4 showing uniformly positive CD20 expression, membranous staining pattern. 6) Excision biopsy of DLBCL (inguinal lymph node) in patient 3 showing uniformly positive CD20 expression, membranous staining pattern.

All biopsied tumor sites showed uptake of ^18^F-FDG and DLBCL localization was confirmed by pathology. IHC was negative for CD20 expression in patient 1 and 6, and positive in the other patients. Patients were ranked based on the intensity level and pattern of CD20 expression (Fig 2).

**Fig 3:**
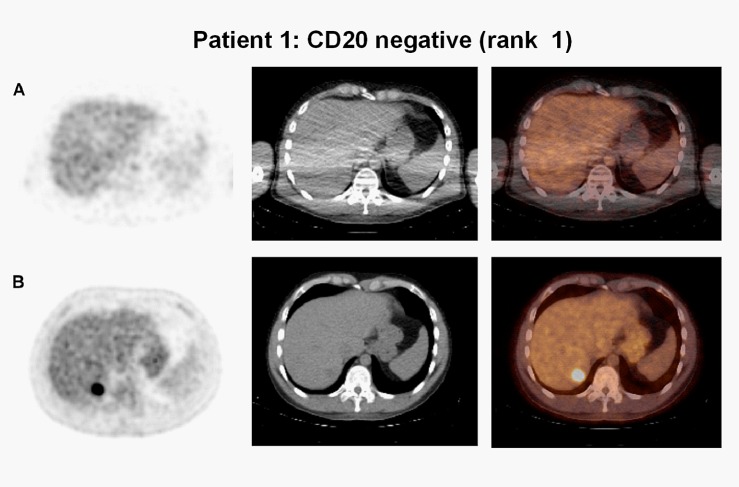
No tumor uptake on ^89^Zr-rituximab-PET, concordant with CD20 expression in biopsy. Axial images, from left to right attenuation corrected PET, low dose CT and fused PET/CT image of patient 1. a) ^89^Zr-rituximab-PET shows no tumor uptake concordant with a CD20-negative liver biopsy. b) Corresponding tumor location on ^18^F-FDG-PET.

**Fig 4:**
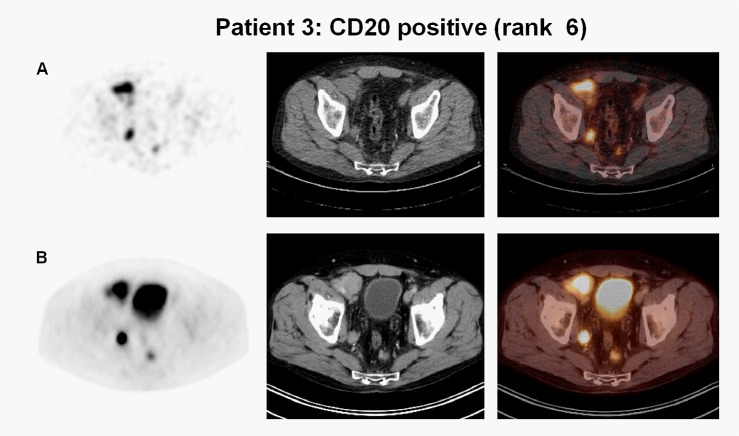
Example of tumor uptake on ^89^Zr-rituximab-PET, concordant with CD20 expression in biopsy. Axial images, from left to right attenuation corrected PET, low dose CT and fused PET/CT image of patient 3. a) ^89^Zr-rituximab-PET shows intense tumor uptake concordant with a CD20-positive biopsy (inguinal lymph node). b) Corresponding tumor location on ^18^F-FDG-PET.

**Fig 5:**
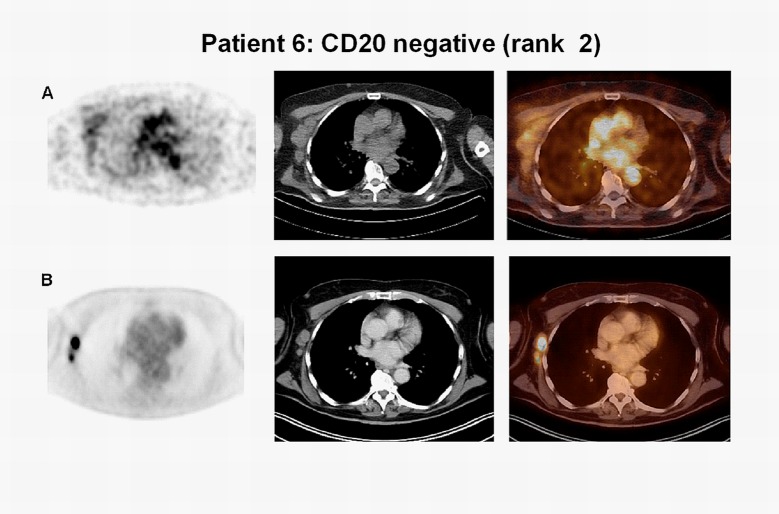
Tumor uptake on ^89^Zr-rituximab-PET, discordant with CD20 expression in biopsy. Axial images, from left to right attenuation corrected PET, low dose CT and fused PET/CT image of patient 6. a) ^89^Zr-rituximab-PET shows tumor uptake discordant with a CD20-negative biopsy (axillar lymph node). b) Corresponding tumor location on ^18^F-FDG-PET.

Tumor uptake of ^89^Zr-rituximab and CD20 expression on IHC were concordant in five patients: for patient 1, both were negative (CD20 rank1, Figs 2 and 3), for the other four patients visible tumor uptake was concordant with CD20-positive biopsies. Intense visual tumor uptake of ^89^Zr-rituximab on PET was observed in patient 3, corresponding with uniformly positive CD20 expression on IHC (Figs 2 and 4). SUV_peak_ for this tumor lesion on D6 was 12.8 (CD20 rank 6). CD20 expression on IHC was also observed in patient 2, 4 and 5 (Fig 2), concordant with tumor uptake of ^89^Zr-rituximab. SUV_peak_ for these tumor lesions on D6 was 3.2, 5.4, 3.4, respectively (CD20 rank 3, 4 and 5, respectively). In one patient (patient 6) tumor uptake of ^89^Zr-rituximab was observed (SUV_peak_ on D6 = 3.8) (Fig 5) while a core needle biopsy was CD20 negative (CD20 rank 2, Fig 2). Tumor uptake over time (SUV_peak_ D6/D3 ratio) was calculated and ranged between 0.6 and 1.4. See Table 3 for PET uptake measures per patient.

**Table 3:** Quantitative tumor uptake measures of ^89^Zr-rituximab in the biopsied tumor lesions.

**Patient**	**CD20 rank**	**Immuno-PET rank**	**SUV**_**peak**_ **D6**	**SUV**_**mean**_ **±SD D6**	**SUV**_**peak**_**/SUV**_**blood**_ **T/B D6**	**SUV**_**peak**_ **D6/D3**	**VOI (mL)**
1	1	1	-	-	-	-	-
2	3	2	3.2	2.3 ± 0.4	NA*	3.2 / 5.3 = 0.6	5.3
3	6	6	12.8	9.1 ± 2.6	12.8 / 2.7 = 4.7	12.8 / 9.1 = 1.4	32
4	5	5	5.4	4.3 ± 0.9	5.4 / 3.4 = 1.6	5.4 / 4.9 = 1.1	7.6
5	4	3	3.4	2.5 ± 0.4	3.4 / 3.1 = 1.1	3.4/ 3.4 = 1.0	25
6	2	4	3.8	3.5 ± 0.5	3.8 / 4.8 = 0.8	NA**	3.5

- = no tumor uptake visible.

NA* = no SUV_blood_ available, only partial scan on D6.

NA ** = no D3 scan available.

A Spearman’s correlation of r_*s*_ = 0.83 and a corresponding p-value of 0.06 were observed between tumor uptake of ^89^Zr-rituximab, measured as SUV_peak_ on D6, and the CD20 expression ranking.

## Discussion

Therapy failure in patients with relapsed/refractory DLBCL may be caused by insufficient CD20-mediated tumor targeting of rituximab. To elucidate the role of tumor targeting, development of an imaging biomarker to assess tumor targeting of rituximab is needed.

To our knowledge, this is the first study to report the performance of ^89^Zr-rituximab-PET as an imaging biomarker for CD20 targeting by correlating tumor uptake of ^89^Zr-rituximab as defined with PET and CD20 expression in biopsied tumor lesions. Tumor biopsies were obtained as routine work-up and tumor uptake on immuno-PET was evaluated at the biopsy sites. Overall, these results suggest a positive correlation between tumor uptake of ^89^Zr-rituximab and CD20 expression in biopsies, but given the small sample size this result should be interpreted with caution. In one patient (patient 6), tumor uptake of ^89^Zr-rituximab was discordant with a CD20-negative biopsy. A possible explanation for the discrepancy is that the tumor site was biopsied in a ^18^F-FDG-PET positive, ^89^Zr-rituximab-PET negative part. IHC is the current gold standard for determination of CD20 expression, however heterogeneity in target expression within and between tumor lesions may not be detected by a single biopsy. Practical limitations of tumor biopsies are the invasiveness of the procedure and the fact that the tumor is not always safely accessible. Another explanation is that measured tumor uptake of ^89^Zr-rituximab is due to blood volume in the tumor, and possibly non-target mediated binding (e.g. to macrophages or neonatal Fc receptor). To our knowledge, there is no literature available that allows to identify the contribution of non-target mediated binding to the PET signal. Tumor to blood ratio’s may give an indication to which extent uptake is higher than can be expected based on blood alone. For the patient with discordant uptake, the tumor to blood ratio on D6 was 0.8, suggesting the possibility that visible tumor uptake could be mainly driven by the blood compartment in the tumor. For the other patients tumor to blood ratio on D6 was ≥ 1.0.

Tumor uptake was quantified in regions with focal uptake exceeding local background. SUV_peak_ is commonly used as a measure of tumor uptake, but reflects only the highest uptake in a small part of the tumor. Manual delineation aims to capture total tumor uptake of ^89^Zr-rituximab, and allows for the derivation of SUV_mean_, its standard deviation and VOI volume. In this study the ranking of PET uptake on D6 was identical for SUV_peak_ and SUV_mean_. For ^18^F-FDG-PET in lymphoma, the Deauville criteria are used to define tumor uptake using liver and mediastinal blood pool as reference region [17]. The observed tumor to blood ratios in this study (range 0.8–4.7) indicate a difference in an uptake criterion based on local contrast versus bloodpool as reference region. To develop a clinically relevant criterion for positive tumor uptake of ^89^Zr-rituximab further studies are required, linking tumor uptake to clinical outcome to rituximab treatment.

A limitation of our study is that the amount of circulating CD20+ B cells, which could influence tracer availability for tumor targeting, was not measured. However, tracer availability in the blood pool could be derived accurately from the image data and was found to be more than 25% ID at D6. By using the blood samples this activity was found to be present in the plasma fraction. Therefore, the presence of a significant CD20 antigen sink hampering tracer availability for tumor targeting can be ruled out in this study.

The use of a predose with unlabeled antibody aims to fill a possible antigen-sink, however recent literature indicates the possibility of saturation of target antigen by unlabeled antibody [8, 18]. Since tumor uptake of ^89^Zr-labeled-mAbs is a slow process taking a considerable amount of time (days), administration of unlabeled (1000mg) and ^89^Zr-labeled rituximab (10mg) can be considered as simultaneous. Therefore, according to the tracer principle, total blocking of tumor uptake by unlabeled antibody is not possible. However, partial saturation with unlabeled antibody can not be excluded and may lower the measured PET signal. The rationale for the study procedure used in this pilot study, including a therapeutic dose of unlabeled rituximab within 2 hours before the ^89^Zr-labeled-rituximab, was to be able to image tumor uptake under conditions that match as closely as possible the therapeutic conditions.

So far, two other clinical studies have been published on the use of ^89^Zr-labeled anti-CD20 with focus on prediction of toxicity for radio-immunotherapy treatment planning in patients with B cell lymphoma [8, 19].

The current study supports further assessment of ^89^Zr-rituximab-PET as an imaging biomarker for CD20 targeting. The patient population in this pilot study was heterogeneous, including differences in age, gender, stage and disease localization at relapse. Also, both primary refractory patients (patient 2 and 6), as well as relapsed patients (patient 1, 3, 4, 5) were included. In this pilot study, no differences were observed in tumor uptake of ^89^Zr-rituximab between patients with primary refractory and relapsed disease. To which extent these factors influence CD20 expression and outcome to rituximab-containing second line treatment remains to be investigated. The observed correlation between CD20 expression and tumor uptake of ^89^Zr-rituximab allows for further studies to assess whether ^89^Zr-rituximab-PET is able to predict which patients will or will not respond to repeated rituximab treatment, and select which patients will benefit from a change of treatment (dose optimization, switch to a different targeted therapy or antibody-drug conjugates (ADC)).

Novel treatment options emerge, including new anti-CD20 mAbs as obinutuzumab and ofatumumab with enhanced capacity for cytotoxicity as well as mAbs for other targets, for instance the anti-CD38 mAb daratumumab, and ADC’s as brentuximab vedotin, an anti-CD30 mAb linked to the antimitotic agent monomethyl auristatin E (MMAE). Molecular imaging with immuno-PET is a promising strategy to guide individualized treatment to improve efficacy, reduce toxicity and costs of mAb treatment.

## Conclusion

This study suggests a positive correlation between tumor uptake of ^89^Zr-rituximab and CD20 expression in biopsies, but further studies are needed to confirm this. This result supports the potential of ^89^Zr-rituximab-PET as an imaging biomarker for CD20 targeting. For clinical application of^89^Zr-rituximab-PET to guide individualized treatment, further studies are required to assess whether tumor targeting is related to clinical benefit of rituximab treatment in individual patients.

## Supporting Information

S1 Fig Total activity (in MBq) in whole blood and plasma on D6. (TIF)
