# Supplementary figures and images for: Performance of 89Zr-Labeled-Rituximab-PET as an Imaging Biomarker to Assess CD20 Targeting: A Pilot Study in Patients with Relapsed/Refractory Diffuse Large B Cell Lymphoma
Source: PLoS One. 2017 Jan 6;12(1):e0169828. doi: 10.1371/journal.pone.0169828 (PMC5218417; doi:10.1371/journal.pone.0169828)

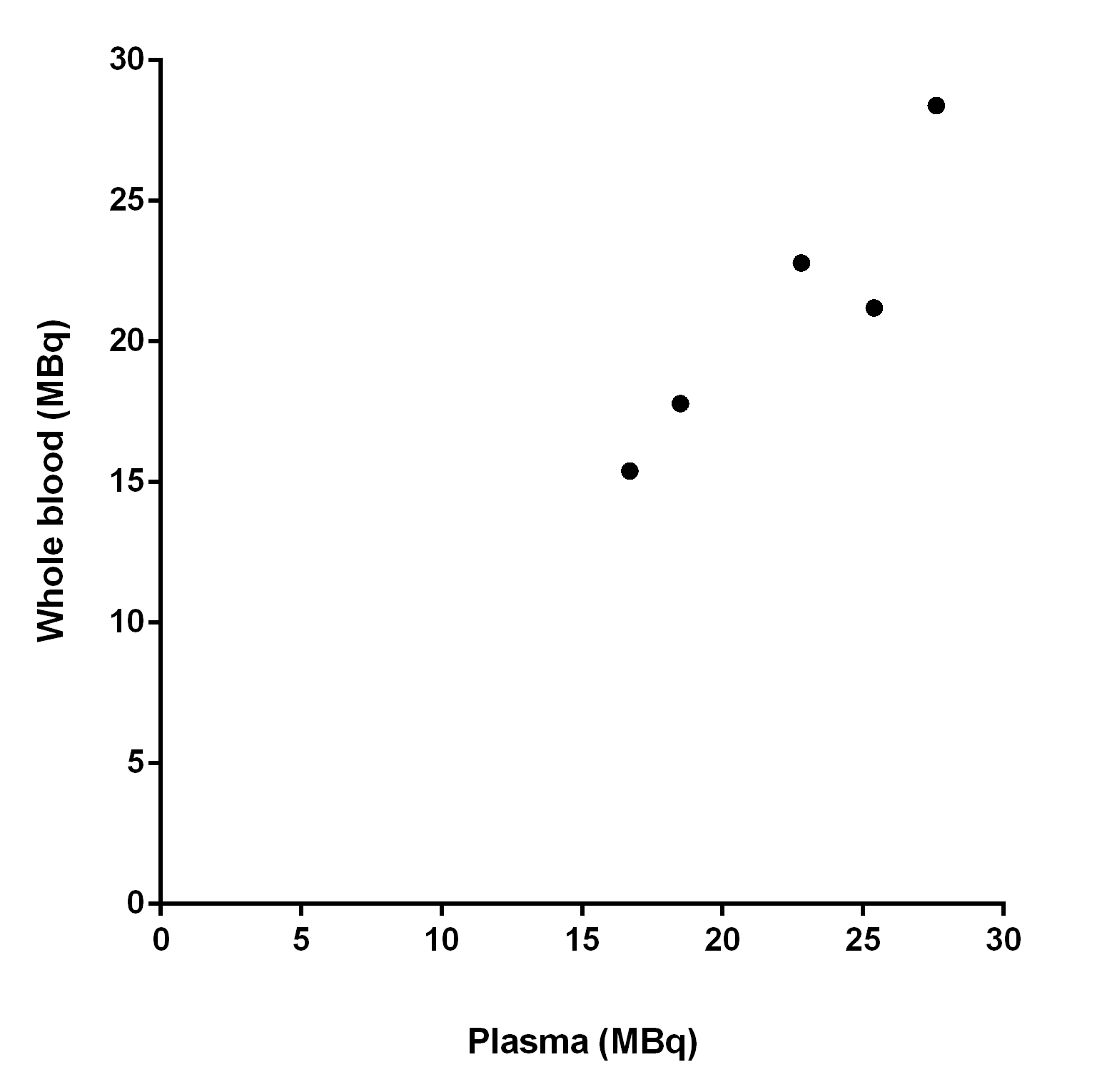

Supplement: S1 Fig — (TIF) [file pone.0169828.s001.tif]
